# Estimating Wolf Population Size and Dynamics by Field Monitoring and Demographic Models: Implications for Management and Conservation

**DOI:** 10.3390/ani13111735

**Published:** 2023-05-24

**Authors:** Enrico Merli, Luca Mattioli, Elena Bassi, Paolo Bongi, Duccio Berzi, Francesca Ciuti, Siriano Luccarini, Federico Morimando, Viviana Viviani, Romolo Caniglia, Marco Galaverni, Elena Fabbri, Massimo Scandura, Marco Apollonio

**Affiliations:** 1 Department of Veterinary Medicine, University of Sassari, 07100 Sassari, Italy; 2 Wildlife Service, Tuscany Region, 50127 Florence, Italy; 3 Unit for Conservation Genetics (BIO-CGE), Italian Institute for Environmental Protection and Research (ISPRA), 40064 Bologna, Italy; 4 Science Unit, WWF Italia, 00198 Rome, Italy

**Keywords:** wolf, population size, population dynamics, demographic drivers, Vortex

## Abstract

**Simple Summary:**

Wildlife conservation effectiveness relies on the accuracy of population data used to build management plans. Estimating population size and dynamics of large vertebrates living on wide areas is a challenging task and results can vary depending on the methods adopted. In the present study, the size of a wolf population was estimated both by field monitoring and statistical modeling on demographic data. Results showed discrepancies with respect to previous bibliographic estimates which resulted significantly lower. Moreover, statistical techniques on demographic data allowed a deeper knowledge of population’s trend, pointing out the importance of mortality rates in driving population growth. We suggest combining statistical modeling and field monitoring to achieve a satisfactory knowledge of population size and dynamics useful to foster reliable management and conservation plans.

**Abstract:**

We estimated the current size and dynamics of the wolf population in Tuscany and investigated the trends and demographic drivers of population changes. Estimates were obtained by two different approaches: (i) mixed-technique field monitoring (from 2014 to 2016) that found the minimum observed pack number and estimated population size, and (ii) an individual-based model (run by Vortex software v. 10.3.8.0) with demographic inputs derived from a local intensive study area and historic data on population size. Field monitoring showed a minimum population size of 558 wolves (SE = 12.005) in 2016, with a density of 2.74 individuals/100 km^2^. The population model described an increasing trend with an average annual rate of increase λ = 1.075 (SE = 0.014), an estimated population size of about 882 individuals (SE = 9.397) in 2016, and a density of 4.29 wolves/100 km^2^. Previously published estimates of wolf population were as low as 56.2% compared to our field monitoring estimation and 34.6% in comparison to our model estimation. We conducted sensitivity tests to analyze the key parameters driving population changes based on juvenile and adult mortality rates, female breeding success, and litter size. Mortality rates played a major role in determining intrinsic growth rate changes, with adult mortality accounting for 62.5% of the total variance explained by the four parameters. Juvenile mortality was responsible for 35.8% of the variance, while female breeding success and litter size had weak or negligible effects. We concluded that reliable estimates of population abundance and a deeper understanding of the role of different demographic parameters in determining population dynamics are crucial to define and carry out appropriate conservation and management strategies to address human–wildlife conflicts.

## 1. Introduction

Wildlife conservation requires accurate knowledge of the key ecological features that affect population dynamics, including population growth and decline [1,2]. Large mammals have complex interactions with their environment [3,4] and they can considerably affect human socio-economic activities, thus requiring integrated management approaches that take into consideration biological, economical, and sociological aspects, relying on scientific evidence [5,6].

The wolf (*Canis lupus*), a species with a complex population dynamic [7] and a potentially severe impact on human activities [8], is a good example of a large carnivore whose presence requires sound management and prompt conservation action, especially due to its recent presence in human-dominated landscapes [9,10] such as in Europe [11]. In these contexts, there is an urgent need to develop a comprehensive approach to address social, emotional, health, and economic aspects related to the conflicts between wolf and human activities [12,13,14,15,16,17,18,19]. Wolf biological populations occur on wide areas (countries and regions), while conflicts often arise and have to be managed at a local scale [20,21]. Acquiring population data needed to properly plan wolf management strategies on a wide geographical scale is challenging. The empirical estimation of wolf population size, dynamics, and demographic parameters needs careful planning, field monitoring, and a combination of complex techniques [7,22]. A promising approach to monitor wolf population size requires a multiple survey method, combining field data with occupancy statistical modeling [23,24]. However, to accurately estimate both population size and trends, a two-spatial scale monitoring approach can also be usefully implemented [25] by monitoring species distribution at a large scale and estimating demographic parameters at a small scale to build population dynamic models, as it has been conducted in Scandinavia (https://www.slu.se/en/departments/ecology/research/teman/wildlife-and-predators-/skandulv/, accessed on 19 November 2022). Likewise, national monitoring programs performed in European countries such as Slovenia [26] and Poland [27] used a common methodology and a coordinated effort to obtain consistent multi-year estimates of population size and dynamics.

Italy has recently completed its first national survey [28] estimating a population size of 3307 wolves (95% CIs = 2945–3608). These results confirm that Italy hosts one of the fastest growing wolf populations in Europe (Boitani and Zimen in 1973 [29] estimated 103 wolves in the Apennines, and the last peer-reviewed published estimate by Galaverni et al. in 2016 [30] reported that the population had grown to 1212 to 1711 animals); however, little is known about actual population trends due to the differences among recent and previous adopted methodologies that are an obstacle to comparisons. For example, in the Italian peninsula, Chapron et al. [11], two years before the previously cited Galaverni et al., estimated a population between 600 to 800 animals (compared to the 1212 to 1711 animals). The first estimate relied mainly on wolf surveys from protected areas in the Northern Apennines published between 2004 and 2012, while the second was obtained by referencing papers published in internationally peer-reviewed journals, public administration reports, unpublished data from the Italian Institute for Environmental Protection and Research (ISPRA), and Standard Data Forms (SDF) completed for the Natura 2000 sites between 2009 and 2013.

To detect population trends, we should also take into account demographic parameters; yet few studies in Italy have focused on mortality, population density, pack density, pack and litter size, breeding success, mate choice, or dispersal rates [31,32,33,34,35,36,37]. Moreover, differences in the monitoring techniques and temporal and spatial scales in these previous studies account for an uncomplete and unbalanced picture of wolf population dynamics. The lack of accurate information on the actual population dynamics and their leading factors prevents a proactive conservation and management of the species useful to foster long-term human–wolf coexistence.

In this case study, we aimed to provide an accurate estimate of the wolf population size in Tuscany, a large Italian region that hosts a high ungulate density [38] and a supposedly large and evenly distributed wolf population. This area presumably acts as a source for the expansion of the species to the Northern Apennine and the Alpine region [32,39,40]. To estimate the minimum number of individuals and packs’ number and location in the region, we combined field data, obtained from wolf-howling [41,42,43] and camera trapping [36,44], with molecular data derived from the microsatellite genotyping of both invasively and non-invasively collected samples [37,44]. Additionally, we performed a Population Viability Analysis (PVA [45]) to build demographic models to better estimate the actual population size, reconstruct previous trends, and predict future population trends. Population viability models, derived from PVA, integrate stochastic and deterministic factors, which influence population dynamics, and they can also quantitatively predict the future status of a population for conservation and management purposes [46]. With all the cautions needed to prevent the misuse of the results [47,48], some studies on wolf dynamics have used PVA to properly determine minimum viable population sizes [49], evaluate control policies [50,51], or plan reintroduction strategies [52]. Despite its effectiveness in comprehensive wolf population dynamics analyses, this approach has been underutilized in the design of conscious and appropriate conservation and management strategies in Italy. To demonstrate the usefulness of such an approach for management purposes, this study, developed at a regional scale, intended to provide insights on southern European wolf population distribution and dynamics. Specifically, we aimed to gain insights of wolf population dynamics and highlight differences in estimates, with respect to field monitoring only, by: (i) estimating the minimum wolf population size in Tuscany by a combination of multiple field survey techniques applied to determine minimum known number of packs and their location; (ii) building an individual-based demographic population model using locally collected demographic parameters to get a more accurate estimation of the actual population size, trends, and the reliability of the ascertained minimum population size, and (iii) determining which demographic parameter (adult mortality, juvenile mortality, female breeding success, or litter size) plays the main role in driving population trends

## 2. Materials and Methods

### 2.1. Study Area

**Figure 1:**
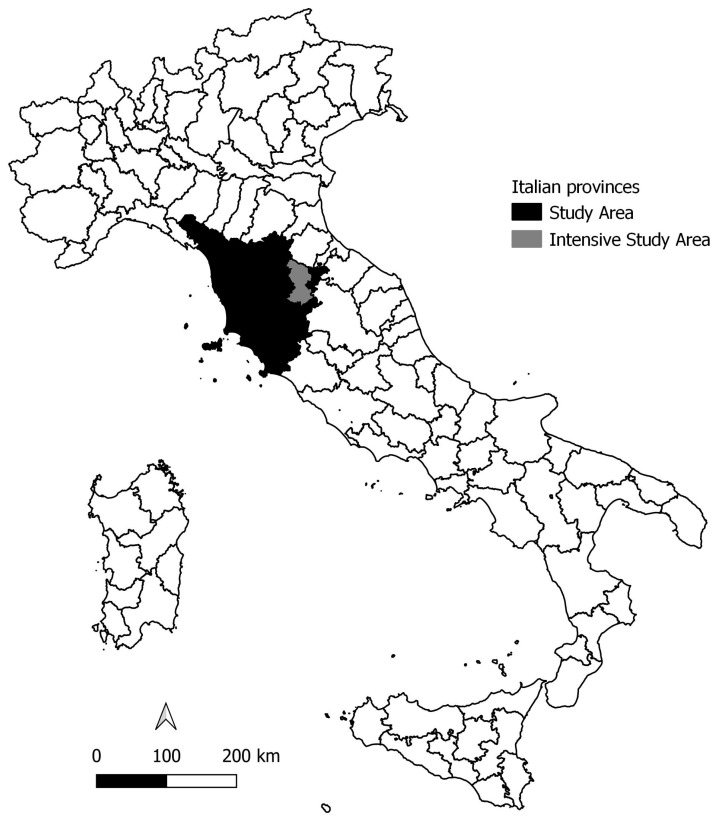
Study Area and Intensive Study Area.

The study was conducted in Tuscany (lat. 43°25′ N, long. 11°00′ E), a region covering about 22,700 km^2^ (excluding islands) located in north-central Italy and placed between the Northern Apennines and the Tyrrhenian Sea (elevations ranging between 0 and about 2050 m a.s.l.) (Figure 1). Most of the territory is hilly (66.5%) or mountainous (25.1%). Forested and bushy areas cover about 11,636 km^2^, corresponding to 50.6%, of the region (Land Use, 2013 [53]) and consist mostly of deciduous woods: manly oak (*Quercus cerris* and *Quercus pubescens*), chestnut (*Castanea sativa*), and beech (*Fagus sylvatica*) in the hilly and mountainous parts of the region and holm oak (*Quercus ilex*) in the littoral area. Conifer woods represent about 15% of the forested areas. About 8765 km^2^ (38.6%) of the region is covered by agricultural areas, mainly grain crops (2000 km^2^), olive groves (920 km^2^), and vineyards (600 km^2^).

Five ungulate species live in Tuscany: red deer (*Cervus elaphus*); fallow deer (*Dama dama*); roe deer (*Capreolus capreolus*) the most abundant species with about 220,000 heads, wild boar (*Sus scrofa*), with about 180,000 heads, and mouflon (*Ovis gmelini musimon*), [54]. Among these, wild boar seems to be the most important prey species for wolves [55,56,57,58,59].

### 2.2. Data Collection and Analysis

Considering that the wolf pack as a social unit is composed of at least one territorial pair [31,36], wolf population monitoring across the whole Tuscan Region was conducted at a large spatial scale between 2014 and 2016 to ascertain the minimum number of packs and their location, regardless of the presence of any offspring in the group. Estimates of demographic parameters were obtained at a small spatial scale and corresponded to a focal area of 560 km^2^ in the Arezzo Tuscan province (Figure 1), where we conducted intensive surveys between 1998 and 2018 to obtain accurate estimates of vital demographic parameters. The consistency of the similarities between the intensive study area and the wolf regional distribution range were evaluated by comparing the habitat characteristics of pack locations inside and outside the area (see Supplementary Materials Figure S2a,b).

#### 2.2.1. Large-Scale Monitoring

On the large scale, we set a monitoring team in each of the 10 administrative provinces in which Tuscany is divided. Each provincial team (hereafter referred to as ‘monitoring unit’ or MU) was led by an experienced coordinator and purposely trained staff. Each MU benefited from having access to previously collected data and a network of local collaborators (i.e., hunters, shepherds, and other volunteers) who provided indications on the presence of putative packs under a contributory project of citizen science approach (sensu Bonney et al. [60]) where all data provided were verified and processed by the MU.

A combination of multiple survey techniques was implemented by MUs [23,61,62,63] to estimate the minimum annual number of packs in the province. Since there were no GPS-collared wolves in the monitored population, the number of packs was obtained by the following approach: (1) ascertaining the presence of packs identified during monitoring activities carried out in the previous years, (2) investigating the presence of new packs in suitable areas not monitored or unoccupied in the previous years, and (3) confirming the presence of packs reported by the citizen science network.

The occurrence of a territorial pack was ascertained by the MU using at least one, but more often a combination, of the following methods: (a) summer wolf-howling survey (in the presence of choral responses with pups and/or two or more adults, according to Apollonio et al. [31]; (b) fixed camera-trapping station survey (in the presence of videos with scent-marking pairs or groups of individuals, or videos with pups); (c) systematic recording of confirmed direct observations of pairs or groups of two or more individuals, or (d) findings of carcasses of reproductive females or pups. Genetic analyses were carried out on non-invasive samples and carcasses to support the identification of local packs. 

Summer wolf-howling surveys were conducted annually between July and October (following Gazzola et al. [41] and Passilongo et al. [42]), but we used an opportunistic approach by focusing on the previously identified or putative packs’ homesites. The sonographic analysis of chorus howls provided information on the number of packs and reproduction events [42,64].

Camera trapping was conducted year-round to confirm pack presence and to collect data on minimum pack size, especially in places where wolf howling was not feasible (i.e., human-dominated landscapes) or ineffective (i.e., no response). Cameras were placed opportunistically to maximize the probability of detection, mainly at scent-marking points such as crossroads, used by wolves along dirt roads [36]. Discrimination of different packs by camera trapping was obtained by combining pack size and composition, occasional natural marks of some individuals, particularly coat color patterns and tail shape and posture during scent-marking display when available [36]. During each annual survey, we considered two adjacent packs as distinct if (a) they were confirmed by wolf howling according to the criteria of Apollonio et al. [31]; (b) they were confirmed as distinct by camera trapping according to Mattioli et al. [36]; (c) one pack was confirmed by wolf howling and the other by camera trapping, they were surveyed in areas >5 km apart, and both had been detected during the previous year, and, (d) one pack was confirmed by wolf howling and the other by camera trapping, they were surveyed in areas >10 km apart, and one or both of them had not been detected during the previous year. 

The values of 5 and 10 km were adopted based on inter-pack distances estimated in previous studies [31,36,65,66] and proved to be consistent with recently published information on the size of the home ranges of the Italian Apennine wolves, which were estimated to average about 104 km^2^ [67], corresponding to a hypothetical circular home range with a radius of 5.75 km. The same 5–10 km spatial criterium was adopted to check for possible double counting by adjacent MUs.

Genetic analyses of carcasses and fecal samples were carried out to obtain individual genetic profiles (i.e., genotypes), based on sets of 11 (feces) or 39 (tissue) autosomal microsatellite loci; match analysis to identify resampled individuals; assignment tests to detect dogs or hybrids, and kinship analysis to reconstruct family units (see Randi et al. [68] and Canu et al. [44] for specific protocols).

All spatial information related to the sampling effort (wolf-howling emission points or camera-trapping stations) and to the data collected to confirm packs (wolf-howling replies, wolf videos, invasive/non-invasive genetic samples, or direct observations) were digitized by MUs using a shared ArcMap [69] environment to avoid double counting of interprovincial packs.

The approximate minimum wolf population size was obtained by adding up the individuals associated with packs with an estimated percentage of lone or dispersing wolves. Wolves in packs were estimated by multiplying the minimum ascertained number of wolf packs by the average pack size detected in the study period by 51 observations of 21 different packs of known size, derived from visual and genetic data, in the intensive study area (4.45, SD = 2.33, see below). Lone wolves accounted for 12.24% (SD = 3.46) of the population. This percentage was estimated by weighting and by sample size, with the values obtained in 11 different studies reviewed by Fuller et al. [7] and Jimenez et al. [62]. The variability of the minimum population size estimates was computed via bootstrap resampling using the “boot” package [70,71] of R statistical software (version 3.4.3 [72]).

#### 2.2.2. Small-Scale Monitoring

Small spatial scale monitoring was performed in the intensive study area using long-lasting genetic analyses from 1998 to 2018, integrated with continuous camera-trapping and howling sessions [37,44,73]. These methods were combined to estimate demographic parameters by collecting data on pack size and composition, litter size, and adult and pup survival based on a sample of 21 packs that were reconstructed at the individual level. A detailed description of the genetic and camera-trapping methodology and wolf individual recognition was reported by Canu et al. [44] and Mattioli et al. [36]. The obtained genotypes were compared with the database of the previously identified wolves and all individual profiles were used for kinship analysis to reconstruct family groups.

Using these long-term studies, we subsequently estimated the following demographic parameters in the intensive study area:

- Maximum age of reproduction was ascertained using known territorial individuals that were genetically recognized across years and were associated to pup presence (by direct observations, camera recording, or howl surveys) within their territory.
- Reproductive success was estimated based on territorial packs and pairs and by considering the lowest estimate from two independent techniques: wolf howling [41,74] and camera trapping. Reproductive success by camera trapping was ascertained only on packs monitored during both summer and winter to confirm pup presence/absence.
- Mean litter size was estimated in summer and in early autumn by camera trapping.
- Juvenile (age < 1 year) mortality was calculated using the difference between summer and late winter litter size. This rate, assuming negligible pup dispersion [75], is an underestimation of true annual mortality, since it does not include deaths in spring and early summer, but its effect on the population model was already considered when estimating the mean litter size, computed in summer, when this mortality already acted.
- Mortality in adults was obtained by finding carcasses of recognized individuals and by camera capture/recapture data on disappearance of reproductive individuals from the camera records (recognized according to Mattioli et al. [36]) and their replacement by other wolves, assuming no pack abandonment by reproductive individuals. Survival differences between females and males were evaluated using the Kaplan–Meier method with the staggered data entry design suggested by Pollock et al. [76].

#### 2.2.3. PVA Model Building

To better describe and analyze population dynamics, we used demographic parameters collected at the small spatial scale to build a PVA model using Vortex software (v. 10.3.8.0) [77]. Vortex PVA integrates, in an individual-based model, the most relevant biological information on a species to give accurate estimates on population trends [78,79,80]. It simulates, for each individual of the population, its life cycle by estimating the outcome of biological events (i.e., reproduction and survival) from probability distribution of demographic parameters, considering stochasticity by replicating the simulation for a high number of times (1000 in the present work). For this purpose, the standard deviations of the weighted averages of demographic parameters were computed via bootstrap resampling (as conducted for the minimum population size) or assumed equal to 25% of the mean if larger, to obtain more conservative forecasts. The PVA model structure accounted for the wolf’s complex social organization and reproductive system, and adhered to Carroll et al. [52], whose model proved to be adequate when describing the life history of the species [48]. Data input and model structure are described in detail in Supplementary Materials Table S2 and Table S3, respectively. The model scenario simulated population growth from a conservative regional estimate of the wolf population in Tuscany since the mid-1980s (derived by combining research estimates from Boitani [81], Ciani [82], and Mattioli and Apollonio [83]. The first year included in the simulation was 1985, when at least 100 wolves were estimated (without adding roaming individuals) to represent the Tuscan population, which was considered isolated for the purposes of this study. Vortex returned the following parameters of interest: (a) population size and trends (estimating both deterministic and stochastic intrinsic growth rate “r”), (b) survival probability of the population, and (c) extinction probability of the population.

#### 2.2.4. Sensitivity Analyses

Sensitivity tests were performed, by generating 100 sets of parameters, to explore the robustness of the population model and the role of some demographic parameters in shaping the population dynamics. Values for four key parameters were drawn from a uniform distribution with a range equal to ±23.6% from the mean value (“relative sensitivity analysis” [84]) of their best estimates. This range was derived from the difference between two robust bibliographical estimations of adult mortality in Italy ([32,35], see below). The four parameters, chosen because of their relation to estimation uncertainty and their known role in population dynamics, were: (i) adult mortality, (ii) juvenile mortality, (iii) female breeding success (percentage of adult females breeding in any year), and (iv) litter size.

Each of the 100 parameter sets was evaluated based on 500 replicate simulations each lasting 31 years (same duration as the original simulation). Using the linear regression of the 4 parameters against the intrinsic growth rate outcomes of the population viability (1), we evaluated the magnitude of the effects of these parameters on the population growth by the amount of variance explained by the models (R^2^), computing the relative importance of three predictor metrics:

(1) r=B1×Adult mortality+B2×Juvenile mortality             +B3×Female breeding sucess+B4×Litter size+ε

where *r* = PVA-estimated intrinsic growth rate; *B*# = estimated regression coefficients; *ε* = model error.

The 3 different metrics, used to evaluate the relative importance of the 4 independent variables (regressors), were computed in order to explore: (i) the percentage of the population growth rate variance explained by each regressor alone (R^2^ first); (ii) the percentage of the population growth rate variance explained by each regressor in addition to all other regressors (R^2^ last), and (iii) the combination of all other available parameters after addressing correlation problems among the predictors (lmg R^2^ decomposition [85,86].Variability of the last metric was assessed via bootstrapping, which allowed for comparisons among predictors. This required the consideration of different models from Equation (1), obtained by recombining the four parameters in all possible ways from a fully saturated model (additive and multiplicative: Adult mortality * Juvenile mortality * Female breeding success * Litter size and all possible decompositions of this quadruple interaction) to single, double, triple, and quadruple parameters for a total of 51 regression models. Sensitivity analysis evaluations were run using the “relaimpo” package [85] of R software (v. 3.4.3), which was also used to run linear regressions. To maximize the final PVA model quality and consistency of results, we followed the assessment framework by Chaudhary and Oli [48], regarding the quality of background information, model structure, and analyses (Supplementary Materials Table S4).

## 3. Results

### 3.1. Large-Scale Monitoring

**Figure 2:**
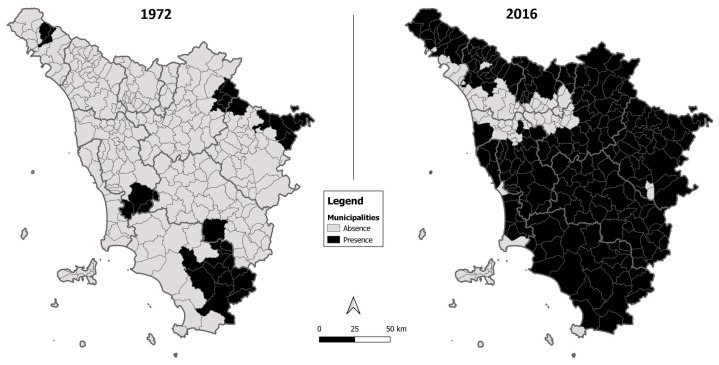
Wolf distribution in the Tuscan municipalities in 1972 (from Cagnolaro et al. [87]) and 2016 (present study).

During the 3 years of integrated monitoring of the wolf population in Tuscany, a total of 3496 observations of wolves or wolf signs were recorded. Of these, 1668 were videos from 28,574 camera-trapping days, across 917 different locations; 213 were responses to 829 howling surveys and 310 were successfully genotyped samples (Supplementary Materials Table S1). These data suggested that from 2014 to 2016, the area continuously occupied by wolves covered at least 222 out of 269 (84.4%) municipalities, an area of about 20,380 km^2^ (89.8% of the region, Figure 2).

Our monitoring efforts confirmed the presence of 135 unique wolf packs. Some of them were observed in more than one year, for a total of 326 pack-years for the whole study period. Specifically: 81 packs (60.0%) were observed during the entire period, 29 (21.5%) across two years, and the remaining 25 were observed in only one year (Supplementary Materials Table S2). We were able to ascertain reproduction in 257 out of these 326 packs, corresponding to 78.8% of the observed packs. Regarding the contribution of different methods in detecting packs, 113 pack-years (34.7%) were ascertained by at least wolf howling, 116 (35.6%) by camera trapping plus other methods but not wolf howling, and the remaining 97 (29.7%) only by other methods, i.e., observation, genetic analysis, and carcasses (Supplementary Materials Table S5). The minimum number of packs increased over the three years of the project, as did the minimum estimated population size, which reached its highest value of 558 wolves in 2016 (Table 1). On average, the minimum estimated wolf density calculated over the three years was 2.43 wolves/100 km^2^ (SD = 0.174) for the whole region and 2.74 wolves/100 km^2^ (SD = 0.19) in the distribution range.

### 3.2. Small-Scale Monitoring

The demographic parameters, estimated from long-term monitoring in the intensive study area, are summarized in Table 2 (Mattioli et al. in prep) and were mostly obtained by camera trapping; 2198 recorded camera capture/recapture data were useful to estimate adult mortality of 35 individually recognized wolves [36], 20 males and 15 females belonging to 11 different packs, observed across a total of 98 wolf-years (on average 22.4 camera records for a wolf each year, SD = 15.95). Among 18 assumed wolf deaths, two cases were directly recorded by finding carcasses.

### 3.3. PVA Model Building

**Figure 3:**
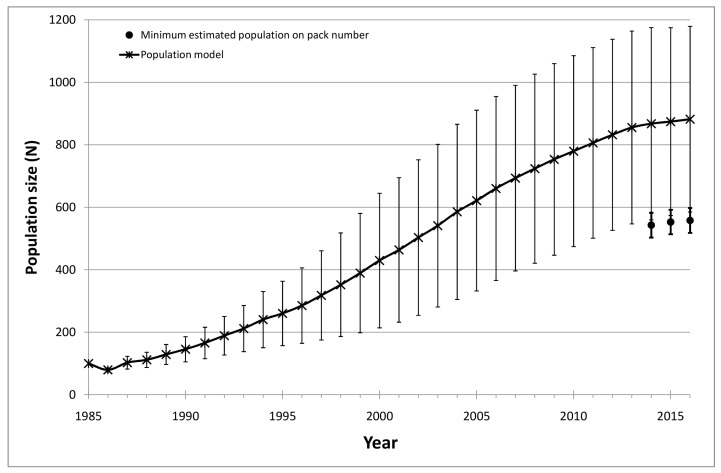
Temporal trends of the wolf population in Tuscany. PVA model estimates (asterisks and continuous line) compared to the minimum population size obtained by field monitoring from 2014 to 2016 (black dots). Standard deviations are indicated by vertical bars.

How the above variables entered the demographic models is detailed in Supplementary Materials Tables S2 and S3. Vortex PVA outcomes predicted a constant increase in population size with an intrinsic growth rate (r) of 0.090 (SE = 0.012) and an average final population size of 882.1 individuals (SE = 9.40) in 2016 (Figure 3). After averaging the final three years of simulated population sizes (874.7 wolves), we estimated a density of 3.85 wolves/100 km^2^ in the Tuscan region corresponding to 4.29 wolves/100 km^2^ in the ascertained distribution range. The probability of extinction was 0.001, with only one occurring in 1000 simulation runs. The finite population annual rate of increase, computed using estimated population sizes over the simulation period, was on average λ = 1.075 (SD = 0.076; min = 0.796; max = 1.289). The comparison of the minimum population size derived by field monitoring in 2014–2016 with the population size predicted by the PVA model showed a constant underestimation by the first approach, which was, on average, 33.75% lower than the latter (Figure 3).

### 3.4. Sensitivity Analyses

**Figure 4:**
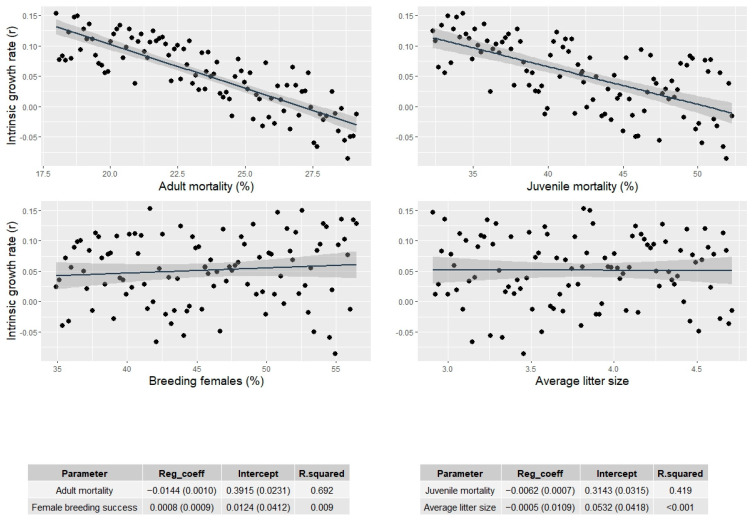
Graphic relationship between the intrinsic population growth rate as estimated by the viability model and the variability of four demographic parameters tested in the sensitivity analysis. Tables show information from the four regression models with each single demographic parameter as a predictor: Reg. coeff. = estimated regression coefficient (SE); intercept = estimated intercept (SE); R-squared = multiple R-squared.

Sensitivity analyses showed that both adult and juvenile mortality played significant roles in the variance of growth rates (Table 3). The percentage of breeding females, which varied between 34.9% and 56.5%, and average litter size, which varied between 2.9 and 4.7 pups, did not show an effect on population trends (regression coefficient B in the single parameter model < 0.001), while the 1% increase of adult mortality caused a decrease of about 0.014 in the intrinsic growth rate (Figure 4).

## 4. Discussion

In this study, the Tuscan wolf population was shown to be larger than previously estimated. It resulted to be, in the study period, a growing population spreading to areas traditionally considered of marginal value for the species [88], but whose suitability should be revised considering the recent population distribution (Figure 2).

Difficulties in monitoring wolves and other carnivores are well known [7,89]. Differences in parameter estimations are expected, depending on the methods used, the ability of monitoring programs to detect key events (such as presence or absence, dispersion, or birth), and the sampling effort expended in the study [11]. Nonetheless, population abundance differences are often too large to be ignored and need to be addressed to reveal the sources of observed variability and to obtain more reliable estimates. Generalizing density data extrapolated from the literature can be inaccurate because of the high heterogeneity observed both in time and space. For example, a review by Chapron et al. [11] estimated an overall European density of 0.94 wolves/100 km^2^, but in the Apennines, such estimates ranged from 1 to 5.2 wolves/100 km^2^ depending on time and areas considered [11,31,32,36,37,90]. The density estimates in this study obtained from field monitoring (2.74 wolves /100 km^2^) and our population model (4.29 wolves/100 km^2^) overlapped with the higher values of the previously cited range for large- and small-scale estimates in Italy and were close to the 5.04 wolves/100 km^2^ estimated by Mattioli et al. [36] in the intensive study area excluding lone transient wolves. These results suggest that wolf density estimates are inversely related to the geographic scale of the study area, which can bias the efficiency of the effort and the consistency of the monitoring scheme throughout the area. Applying a consistent monitoring effort to all regional territories (see Supplementary Files) significantly increased the estimate of Tuscany’s wolf population size previously derived by extrapolation of bibliographic data. As an example, the published estimation of the Tuscan wolf population in the same years of our study was provided by Galaverni et al. [30] averaging 305 wolves, which represent 56.2% of our minimum population size (543 wolves) estimated by field work in 2014.

Even when the goal of wolf surveys is achieving accurate estimations of the number of packs and breeding units [61], field monitoring is generally incomplete, as some packs are missing. Marucco et al. [35] found that snow tracking in the Italian Alps estimated 36.2% fewer wolves than the number estimated using capture–recapture modeling on the same population. In the Northwest Iberian Peninsula, Jimenez et al. [62] found that howling surveys identified only 49.8% of the reproductive units estimated by occupancy models, while in Idaho (U.S.A.), official minimum counts derived by combining data from multiple empirical surveys resulted to be 85.7% of the wolves estimated by occupancy models by Ausband et al. [23].

In our study, the average minimum population size empirically estimated from 2014 to 2016 corresponded to 66.2% of the number estimated by the population viability model, which predicted around 882 wolves in Tuscany in 2016 with good precision (SE = 9.40) and a standard deviation (297.17) of the same magnitude of the variability in parameters’ estimates. This result was consistent with other long-term monitoring efforts, such as those in some North American states (e.g., Montana, Idaho, and Wisconsin) where wolves have experienced a recovery pattern as in Italy. In these states, monitoring procedures combined the minimum wolf population based on pack recordings with other statistical approaches [91]. This was the case in Montana [92] and Wisconsin [63], where the verified minimum number of residing wolves was followed and combined with a patch occupancy model to estimate the actual number of wolves. Similarly, we consider our empirical estimates, falling within the lower limits of the model’s confidence intervals, an underestimation of the true population size. Our model results, in turn, can be evaluated as an underestimation of the real biotic potential of the population, since considering a close population, as conducted in the present study, excluded the contribution from neighboring populations that can be a major component of population increase [7]. Population size can be better estimated by combining monitoring approach, which can obtain a precise knowledge of demographic parameters from an adequate number of resident packs, with the modeling approach, thus providing a more realistic estimation of the population size.

For what concerns population dynamics, our Vortex model described, as expected, a growing population with an average finite growth rate λ = 1.08, in the years 2014–2016, like almost simultaneous estimates in the nearby Northern Apennines (1.05 [32]) and in the Alps (1.04 [35]). Population finite growth rate has been reported at higher values elsewhere, possibly due to different ecological conditions (prey abundance, pack size, human density) or to different position with respect to the exponential growth model curve, so that a better comparison should be conducted with intrinsic growth rates, not available from other Italian studies. The resulting Tuscan population size and distribution, widespread throughout the region, was not limited to mountain-forested environments as predicted by previous environmental suitability models [93], suggesting the need for more up-to-date habitat suitability models and monitoring and management strategies.

A population model’s predictive accuracy depends on the key parameters used and on the reliability of environmental conditions of the simulations [47,80]. The key demographic parameters used in this study to build the population model were breeding success (imputed in the model as the percentage of adult breeding females), litter size (in summer), and adult and juvenile survival. Breeding success is usually estimated as the percentage of reproductive packs [92]. A previous study in the Foreste Casentinesi National Park [31] recorded 78% of packs with litters, very similar to the results of this study, where the observed percentage of reproductive packs varied from a minimum of 73.1% (by camera trapping) to a maximum of 82.4% (by wolf howling). These results differ from what was observed in North America, where most packs produce pups every year [7]. This difference could be partly due to the methodology adopted to assess the parameter, that in our study relied on the observation of pups in summer, when pup mortality partially already occurred, while other studies rely on observations in spring at the den or on radio-collared wolves [94] or on the analysis of placental scars [95]. However, much scarcer estimates have been found for the percentage of breeding females (the parameter needed by Vortex, which cannot consider pack reproductive success). Although this information was not available for the Italian wolf population, the values were highly variable across Europe and North America, ranging from a minimum value of 29% of breeding adult females in the Scandinavian population [50], to 38% in Yellowstone [96], and up to 50–64% in Ontario [97]. In the intensive study area in Arezzo, 45.6% of adult females were observed to breed, which was close to the 50% found by Carroll et al. [52] when they simulated the viability of a Mexican wolf population reintroduced to the Southwestern United States.

Litter size can be determined at the den or later in the summer, when juvenile mortality has partially already occurred, which reduces the number of pups that can be detected. Previous studies reported 4.88 to 6.9 pups per litter at the den [22,50,52], while litter size in summer to autumn was estimated to range from 2.2 to 3.4 pups [31,65,98,99]. The average litter size estimated in our focus area was higher, around 3.9 pups, but with a large SD (1.97) and a range of 1 to 7 pups. This high variability was not surprising because of the differences in juvenile mortality that can be more relevant in either the first six months of life [35] or in the second six months [100], depending on environmental conditions.

The annual mortality rate can be highly variable for pups, as reported by Smith at al. [100] who studied three different North American wolf populations and recorded pup mortality values between autumn and spring ranging from 11.1% to 60.2%. In Europe, the annual juvenile mortality estimates ranged from a minimum of 22% in [50] to a maximum of 76% in the Italian Alps [35]. Our estimate of 42.3% falls in the middle of this range but should be referenced with caution because of parameter variability and the small sample size. Adult mortality was found to be 20.4%, within the range of the two previous estimates [32,35], but also very similar to the average found in North America (and particularly to that found in the Superior National Forest, Minnesota [101]), despite the great variability of this value [7,102]. Our sensitivity analysis pointed out that both adult and juvenile mortality play primary roles when determining population fate. This finding was consistent with those of Heppel et al. [103] and van de Kerk et al. [104], who found that mortality rates were important when determining the growth rates of mammals and carnivores with medium-sized body mass and long life spans and generation times. The importance to invest in accurate estimation of mortality rates was fostered by the reduced effect of female breeding success and average litter size on the midterm previsions of population trends, bypassing wolf howling difficulties to gain precise estimates of a pack’s reproduction [42,64].

From a management point of view, the availability of more accurate data on wolf population size, distribution of packs, and trends is fundamental to properly plan species conservation. Our study provided a new estimate of the population that justify a switch in action priorities. Tuscan wolf population looked more abundant and healthier (growing) than previously described, thus conservation strategies and resources could focus on conflict reduction rather than acting on environmental limiting factors (i.e., habitat suitability or human disturbance). Large predators are more tolerated by stakeholders when people are provided with correct information about benefits in coexistence and about techniques to reduce risks [13]. It has been observed that the positive attitude of people towards wolves decreases with the decrease of the distance from packs [105] and the social intolerance of wolves together with reduced trust in authorities can foster poaching, limiting species recovery [16]. In the light of the updated wolf population status (i.e., size and distribution), policy priorities need to be revised, providing effective measures to prevent livestock depredation, and to make people aware both in rural- and human-dominated landscapes.

## 5. Conclusions

In conclusion, our case study showed a large discrepancy between the previous and the present abundance estimates of the wolf population living in the Tuscany region. This gap is even bigger if we consider our results as underestimations of the real size of the population, whose positive status and growing trend had been ignored so far. This should encourage regular replicates of national and local estimates derived from the actual location of a representative number of known packs. Our results highlighted the need to achieve robust estimates of demographic parameters, particularly mortality rates, to implement sound population models that can be useful for appropriate conservation and management planning. It is particularly relevant to combine empirical estimations of demographic parameters with population modeling, as the absence of one of these factors could result in the absence of reliable estimations of population status and trends.

Consequently, the lack of such data on the Italian wolf population, one of the fastest increasing wolf populations in southern Europe [11], makes unreliable previous existing abundance estimates.

The wolf elicits attention and contrasting attitudes given its increasing presence in human-dominated landscapes [11,16]. The complexity of its ecology (see, for instance, the effect of breeder mortality on population dynamics as highlighted by Brainerd et al. [106] and Borg et al. [107]) requires careful and conscious management approaches that rest on scientific evidence and proper techniques obtained from the same populations intending to be managed and conserved.

## Figures and Tables

**Table 1:** Minimum number of observed packs and estimated wolf population size in Tuscany.

Year	Minimum Number of Observed Packs	Average Pack Size	% of Non-Territorial Wolves	Minimum Estimated Wolf Population
2014	107	4.45 (SD = 2.33)	12.24 (SD = 3.46)	543 (SD = 39.75)
2015	109			553 (SD = 39.11)
2016	110			558 (SD = 39.82)

**Table 2:** Average demographic parameters, Annual Standard Deviation (SD), Variation range (Min–Max), and sample size resulting from the long-term monitoring scheme in the intensive study area.

Parameter	Method	Value	SD	Min–Max	Sample Size
Maximum age of reproduction	Genetics	7 y males 10 y females	- -	2–7 2–10	1 on 8 females 1 on 11 males
Pack reproductive success	Camera trapping	73.07%	21.75	0–100	52 pack-years (14 packs)
	Wolf howling	82.40%	10.62	60–100%	69 pack-years (9 packs)
Female reproductive success	Camera trapping	45.59%	18.43	0–100%	68 adult females (8 years)
Summer average litter size	Camera trapping	3.81	1.97	1–7	21 litters (8 packs)
Juvenile mortality (from summer to spring)	Camera trapping	42.31%	10.58	0–100%	52 pups, 16 litters (6 packs)
Adult mortality	Camera trapping	20.41%	5.01	0–29%	35 wolves (98 occasions)

**Table 3:** Variability of the demographic parameters tested in the sensitivity analysis and relative importance in explaining the variance of the population intrinsic growth rate.

Parameter	Min	Max	Relative Importance Metrics				
			R^2^ First	R^2^ Last	LMG R^2^ Decomposition		
					R^2^ Contribution	Lower 95% C.I.	Upper 95% C.I.
Adult mortality (%)	18.00	29.50	0.692	0.553	0.625	0.560	0.679
Juvenile mortality (%)	32.32	52.29	0.419	0.292	0.358	0.290	0.412
Percentage of breeding female	34.91	56.48	0.009	<0.001	0.005	0.001	0.039
Average litter size	2.91	4.71	<0.001	<0.001	0.002	0.000	0.026
Total R^2^ of the fully saturated linear model = 0.989							

## Data Availability

Detailed data of field monitoring efforts and results are provided in Supplementary Materials Table S1. Model inputs are provided in Supplementary Materials Table S2. Model structure and parameters are provided in Supplementary Materials Table S3. Raw data for sensitivity analysis were provided in the file: ‘lupo_toscana_ST_mar2020_4var.csv’.

## Supplementary Materials

The following supporting information can be downloaded at: https://www.mdpi.com/article/10.3390/ani13111735/s1, Figure S1: Overall packs’ location and persistence; Figure S2: Visual comparison of the environmental characteristics between inside and outside intensive study area (a) Relative importance of habitat types in a 104 km^2^ buffer around packs’ locations; (b) Mean weighted elevation of a 104 km^2^ buffer around packs’ locations; Figure S3: Visual comparison among estimates of population trends by demographic models with different initial population size; Table S1: Detailed effort and results in regional integrated survey; Table S2: Demographic input values and specifications for Vortex analysis. Table S3: Vortex PVA State variables and model structure. Table S4: Self-assessment of the PVA model quality by means of the guiding questions of Chaudhary and Oli (2020). Table S5: Frequency of yearly success of packs detection methods. References [35,48,49,52,54,81,82,83,102,108,109,110,111,112,113,114,115] are cited in the supplementary materials.
