# Supplementary material for: Estimating Wolf Population Size and Dynamics by Field Monitoring and Demographic Models: Implications for Management and Conservation
Source: Animals (Basel). 2023 May 24;13(11):1735. doi: 10.3390/ani13111735 (PMC10252110; doi:10.3390/ani13111735)
Supplement: Supplementary file 1 [file animals-13-01735-s001.zip › Table S1.pdf]

SUPPLEMENTARY MATERIAL

Table S1

Detailed effort and results in regional integrated survey

Provinces' acronyms: AR=Arezzo (including intensive study area), FI=Firenze, GR=Grosseto, LI=Livorno, LU=Lucca, MS=Massa-Carrara, PI=Pisa, PO=Prato, PT=Pistoia, SI=Siena; Wolf-howling: sessions =number of nights spent to stimulate howls from multiple points in the same area, positive responses= nights recording wolves' howls; Camera trapping: Sessions = number of trapping days, Locations = clusters of camera traps (within 3 km radius), Positive responses = records of wolves (every ten minutes). Packs detected by each Monitoring Unit, in bracket n° of packs occupying a territory in two or more different provinces (detected packs sum doesn't return the minimum number of observed packs).

**Province**	**Year**	**Wolf howling**		**Camera trapping**			**Genetic monitoring**		**Packs detected (shared)**
		**Sessions**	**Positive responses**	**Sessions**	**Locations (N)**	**Positive responses**	**Samples collected**	**Positive responses**	
AR	2014	24	12	3043	41	536	44	30	22 (4)
FI	2014	24	10	1170	62	41	1	1	19 (7)
GR	2014	27	4	510	34	26	2	2	21 (1)
LI	2014	4	1	600	8	30	0	0	2 (1)
LU	2014	12	3	480	8	6	1	1	9 (5)
MS	2014	57	9	240	12	7	1	1	8 (2)
PI	2014	18	3	815	12	20	1	1	7 (5)
PO	2014	9	5	600	20	16	0	0	4 (3)
PT	2014	17	3	552	24	16	8	8	12 (5)
SI	2014	30	8	600	40	29	17	17	22 (4)
2014 minimum regional number of observed packs (N° of packs shared by provinces)									**107 (18)**
AR	2015	28	9	5119	79	1125	94	84	25 (4)
FI	2015	26	11	1220	68	46	7	4	18 (6)
GR	2015	80	25	1050	70	49	42	30	16 (1)
LI	2015	19	1	505	7	37	4	3	3 (1)
LU	2015	14	5	600	18	10	3	2	9 (4)
MS	2015	64	11	480	24	14	4	4	8 (1)
PI	2015	10	2	920	13	35	14	1	8 (5)
PO	2015	12	4	640	24	18	1	0	4 (3)
PT	2015	27	8	441	16	12	23	20	12 (5)
SI	2015	21	7	510	34	27	1	1	24 (5)
2015 minimum regional number of observed packs (N° of packs shared by provinces)									**109 (17)**
AR	2016	10	5	2144	36	611	66	41	25 (2)
FI	2016	35	12	1200	58	32	15	9	23 (4)
GR	2016	80	22	900	60	38	23	10	16 (0)
LI	2016	20	2	480	9	66	10	2	3 (1)
LU	2016	18	4	720	22	12	8	6	8 (2)
MS	2016	62	8	480	24	11	7	2	8 (1)
PI	2016	22	4	1080	24	40	19	16	5 (1)
PO	2016	15	4	720	26	20	1	1	5 (3)
PT	2016	24	6	305	14	11	11	7	9 (3)
SI	2016	20	5	450	30	26	13	6	17 (1)
2016 minimum regional number of observed packs (N° of packs shared by provinces)									**110 (15)**
Yearly regional mean (min-max)		276.3 (222-306)	71.0 (58-83)	9524.7 (8610-11485)	305.7 (261-353)	989.0 (727-1373)	147.0 (75-174)	105.3 (61-149)	**108.7 (107-110)**
