# Supplementary material for: Estimating Wolf Population Size and Dynamics by Field Monitoring and Demographic Models: Implications for Management and Conservation
Source: Animals (Basel). 2023 May 24;13(11):1735. doi: 10.3390/ani13111735 (PMC10252110; doi:10.3390/ani13111735)
Supplement: Supplementary file 1 [file animals-13-01735-s001.zip › Table S2.pdf]

SUPPLEMENTARY MATERIAL

Table S2

Demographic input values and specifications for Vortex analysis

Model structure was derived from Carroll et al. 2013, with Vortex run as an Individual Based Model and enabling Special Options, in order to follow complex biology of the species. Since the aim of the present study was to model the actual increase of Tuscan wolf population, rather than to foresee future development of a reintroduced population, we considered a single population rather than a meta-population and without any genetic management of mating as done, instead, by Carrol et al. [52].

Parameter	Value (SD)	Notes
Initial population size	100	This value was obtained by summarizing estimates from different Tuscan areas of Boitani et al. [81] (30-50), Ciani [82] (20-25 wolves) and Mattioli and Apollonio [83] (50-100). This value was considered as the most reliable conservative estimation, relying on a minimum ascertained number of individuals, but uncertainties on the parameter were explored varying simulation inputs without substantially affecting the results (see Figure S3).
Inbreeding depression, lethal equivalents	6.29	Program default values (by O'Grady et al. [108]). Since wolf population was strongly increasing, we didn't deeply investigate inbreeding depression genetic implications, but in any case, we didn't exclude effects in the few occasions when population stochastically dropped
Percent due to lethal recessive alleles	50	
Reproductive system	Long term monogamy	As done by Carroll et al. [52], we incorporated into the model the persistent monopolization of breeding opportunities by male and female alpha individuals. Once an individual achieves alpha status it will generally retain that status until death
Age of first offspring females	2	
Maximum age of female reproduction	10	Observed in the focus area by combining evidences of reproduction (direct observations, camera-trapping records or wolf-howling) with genetic information
Age of first offspring males	2	
Maximum age of male reproduction	7	Observed in the focus area
Maximum lifespan	11	Observed in the focus area
Maximum number of broods per year	1	Observed in the focus area
Maximum number of progeny per brood	7	Observed in the focus area
Sex ratio at birth in % males	50	
Density dependence	no	Since population size was far from estimated carrying capacity (see parameter explanation) for most of the study period, we were not able to observe density dependence. Hence, we assumed no such a link, in order to produce more conservative predictions [48,109]. Moreover, no dead wolves were found during the monitoring period due to intraspecific aggression, that was

		found to be, at least in some circumstance, a density-dependent regulator of adult mortality [102].
% adult females breeding	45.588 (18.429)	The value was imputed in the model by typing: 100*IS7 (See State variables, Tab. S3), in order tobetter represent wolf complex reproductive system, as done by Carrol et al. [52].<br>The parameter was obtained by monitoring 19 packs for 56 reproduction occasions with 68 adult females in the focus area. In 31 occasions pups were recorded in summer.
Mean number of offspring per female per brood	3.81 (1.965)	Mean summer litter size from 21 observations of 10 different packs. This is an underestimation of the true brood size, since it accounts for the first part of juvenile mortality, but the estimation of this kind of mortality was adjusted in population model in order to exclude the quota already computed in the brood size (See next).
% Juvenile mortality	42.31 (10.58)	Juvenile mortality was derived as the percentage of difference between summer and late winter brood size. This mortality constitutes an underestimation of the true annual juvenile mortality, since it doesn't include spring and early summer mortality, which can be relevant [35], but whose effect, in the population model, was already considered in the mean brood size.<br>SD was assumed to be 25% of the average value.
% Adult mortality	20.41 (5.01)	The value was imputed in the model by typing: =PS5 (See State variables, Tab. S3), in order to better represent wolf complex reproductive system, simplifying Carrol et al. [52].<br>It was estimated from 6 years monitoring, 98 occasions (52 for males, 46 females), on 35 different individuals (20 males and 15 females).<br>SD was assumed to be 25% of the average value
Catastrophes	0	Since there were no evidences of catastrophic events in the study period to estimate reliable occurrence rates for catastrophes and their incidence on population [49]
% Males in the breeding pool	100*IS8	The value was computed from the number of adult males alive and the number of breeding females. The parameter was imputed in the model by mean of state variables as done for females and similarly to Carroll et al. [52], simplified (See State variables Tab. S3).
Carrying capacity	1466.8 (60.2)	The maximum carrying capacity was defined assuming it depended almost on available wild ungulates biomass [110]. Ungulates populations were estimated by hunting censuses in 2016 [54] and assuming an annual intake rate of 972 kg as the need for a wolf [111]. Available biomass was computed subtracting the hunting quota and by assuming:<br>20 kg for each roe deer;<br>100 kg for each red deer;

		50 kg for each fallow deer;<br>30 kg for each mouflon;<br>30 kg for each wild boar.<br>See also Person et al. [112]; Karlsson et al. [113]; Fuller & Keith [114]; Cariappa [115].<br>Since observed ungulate abundance is lower in most anthropized areas, the parameter reduces, accordingly, the overall estimated wolf density with respect to the contribution provided by hilly and mountainous areas

References

35. Marucco, F.; Pletscher, D.H.; Boitani, L.; Schwartz, M.K.; Pilgrim, K.L.; Lebreton, J.-D. Wolf survival and population trend using non-invasive capture-recapture techniques in the Western Alps. *J. Appl. Ecol.* **2009**, *46*, 1003–1010. https://doi.org/10.1111/j.1365-2664.2009.01696.x.
48. Chaudhary, V.; Oli, M.K. A critical appraisal of population viability analysis. *Conserv. Biol.* **2020**, *34*, 26–40. https://doi.org/10.1111/cobi.13414.
49. Chapron, G.; Andrèn, H.; Sand, H.; Liberg, O. *Demographic Viability of the Scandinavian Wolf Population. A Report by SKANDULV*; Swedish Environmental Protection Agency: Riddarhyttan, Sweden, 2012; 56p.
52. Carroll, C.; Fredrickson, R.J.; Lacy, R.C. Developing Metapopulation Connectivity Criteria from Genetic and Habitat Data to Recover the Endangered Mexican Wolf. *Conserv. Biol.* **2013**, *28*, 76–86. https://doi.org/10.1111/cobi.12156.
54. Tuscany Region. L'Attuazione della l.r. 10/2016 "Legge Obiettivo per la Gestione Degli Ungulati in Toscana". Nota Informativa Sull'Attuazione delle Politiche Regionali (Unique Volume). 2018, 1–12. https://www.consiglio.regione.toscana.it/upload/COCCOINA/documenti/nota%20informativa%2040_pub.pdf (accessed on 20 November 2022). (In Italian)
81. Boitani, L.; Ciucci, P.; Corsi, F.; Fabbri, M.L. Assessing actual and potential wolf range in Italy with the aid of a GIS. In Pro-ceedings of the 5th International Theriological Conference, Rome, Italy, 22–29 August 1989.
82. Ciani, F. Attuale situazione del Lupo nell'Appennino toscano, tendenza del popolamento negli ultimi 10 anni. In Proceedings of the Atti Convegno Nazionale Straordinario del Gruppo Lupo Italia, Arcidosso, Italy, 28–30 September 1990. (In Italian)
83. Mattioli, L.; Apollonio, M. Status del lupo in Provincia di Arezzo 2012–2013. Report Annuale per CIRSEMAF-Regione Toscana; Regione Toscana: Firenze, Italy, 2013. (In Italian).
102. Cubaynes, S.; MacNulty, D.R.; Stahler, D.R.; Quimby, K.A.; Smith, D.W.; Coulson, T. Density-dependent intraspecific aggression regulates survival in northern Yellowstone wolves (*Canis lupus*). *J. Anim. Ecol.* **2014**, *83*, 1344–1356. https://doi.org/10.1111/1365-2656.12238.
108. O'grady, J.J.; Brook, B.W.; Reed, D.H.; Ballou, J.D.; Tonkyn, D.W.; Frankham, R. Realistic levels of inbreeding depression strongly affect extinction risk in wild populations. *Biol. Conserv.* **2006**, *133*, 42–51. https://doi.org/10.1016/j.biocon.2006.05.016.
109. Ginzburg, L.R.; Ferson, S.; Akcakaya, H.R. Reconstructibility of Density Dependence and the Conservative Assessment of Extinction Risks. *Conserv. Biol.* **1990**, *4*, 63–70. https://doi.org/10.1111/j.1523-1739.1990.tb00268.x.
110. Mcroberts, R.E.; Mech, L.D. Wolf population regulation revisited-Again. *J. Wildl. Manag.* **2014**, *78*, 963–967. https://doi.org/10.1002/jwmg.744.
111. Gazzola, A.; Bertelli, I.; Avanzinelli, E.; Tolosano, A.; Bertotto, P.; Apollonio, M. Predation by wolves (*Canis lupus*) on wild and domestic ungulates of the western Alps, Italy. *J. Zoöl.* **2005**, *266*, 205–213. https://doi.org/10.1017/s0952836905006801.
112. Person, D.K.; Kirchhoff, M.; Van Ballenberghe, V.; Iverson, G.C.; Grossman, E. *The Alexander Archipelago Wolf: A Conservation Assessment*; U.S. Department of Agriculture, Forest Service, Pacific Northwest Research Station: Portland, OR, USA, 1996; PNW-GTR-384. https://doi.org/10.2737/pnw-gtr-384.
113. Karlsson, K.; Brøseth, H.; Sand, H.; Andre´n, H. Predicting occurrence of wolf territories in Scandinavia. *J. Zoöl.* **2007**, *272*, 276–283. https://doi.org/10.1111/j.1469-7998.2006.00267.x.
114. Fuller, T.K.; Keith, L.B. Non-Overlapping Ranges of Coyotes and Wolves in Northeastern Alberta. *J. Mammal.* **1981**, *62*, 403–405. https://doi.org/10.2307/1380725.
115. Cariappa, C.A.; Oakleaf, J.K.; Ballard, W.B.; Breck, S.W. A reappraisal of the evidence for regulation of wolf populations. *J. Wildl. Manag.* **2011**, *75*, 726–730. https://doi.org/10.1002/jwmg.74.
