# Supplementary material for: Estimating Wolf Population Size and Dynamics by Field Monitoring and Demographic Models: Implications for Management and Conservation
Source: Animals (Basel). 2023 May 24;13(11):1735. doi: 10.3390/ani13111735 (PMC10252110; doi:10.3390/ani13111735)
Supplement: Supplementary file 1 [file animals-13-01735-s001.zip › Table S3.pdf]

SUPPLEMENTARY MATERIAL

Table S3

Vortex PVA State variables and model structure

Population state variable ID	Initialization function	Transition function
PS1	=(45.588+(18.429*NRAND))/100	=(45.588+(18.429*NRAND))/100
PS2	=ITOT3	=ITOT3
PS3	=ITOT4	=ITOT4
PS4	=ITOT6	=ITOT6
PS5	=(20.41+(5.01*NRAND))	=(20.41+(5.01*NRAND))

Individual state variable ID – Label	Initialization function	Birth function	Transition function
IS1 – MATE	=-1	=-1	=IS1
IS2 – PREVBRDR	=IS3	=0	=IS3
IS3 – BRDR	=(IS1>0)	=0	=(NMATES>0)
IS4 – FBRDR	=(S='F')*IS3	=0	=(S='F')*IS3
IS5 – MBRDR	=(S='M')*IS3	=0	=(S='M')*IS3
IS6 – ADULTF	=(S='F')*(A>0)	=0	=(S='F')*(A>0)
IS7 – FPOOL	=(IS4=1)	=0	=(S='F')*(A>1)*(A<11)*[(ROUND(PS1*PS4)<PS3)*((IS3=1)OR((IS3=0)AND(IS2=1)))*(((PS1*PS4)/PS3)>=RAND)+(ROUND(PS1*PS4)=PS3)*((IS3=1)OR((IS3=0)AND(IS2=1)))+(ROUND(PS1*PS4)>PS3)*[(IS3=1)+(IS3=0)*(IS2=1)+(IS3=0)*(IS2=0)*((((PS1*PS4)-PS3)/(PS4-PS3))>RAND)]]
IS8 – MPOOL	=(IS5=1)	=0	=(S='M')*[(IS3=1)OR(A<7)*(IS3=0)*(IS2=1)OR(IS3=0)*(IS2=0)*(A>1)*(A<7)*[((ROUND(PS1*PS4))<(PS2-PS3))*{(([(2*PS5*PS3)+((PS1*PS4)>PS3)*{2*((PS1*PS4)-PS3)}-{(PS2-PS3)-(0.71*PS3)}]/{(M+0.01)-(PS2-PS3)})>=RAND}+((ROUND(PS1*PS4))=(PS2-PS3))*((1.2*PS5*0.01*(PS2-PS3)}/{(M+0.01)-(PS2-PS3)})>=RAND)+((ROUND(PS1*PS4))>(PS2-PS3))*(({(2*((PS1*PS4)-(PS2-PS3)))+(1.2*PS5*(PS2-PS3))}/{(M+0.01)-(PS2-PS3)})>=RAND)]]

IS7 and IS8 were built as done for IS22 and IS23 respectively, in Carrol et al. [52], describing “probability to breed” computation for adult females and males

References

52. Carroll, C.; Fredrickson, R.J.; Lacy, R.C. Developing Metapopulation Connectivity Criteria from Genetic and Habitat Data to Recover the Endangered Mexican Wolf. *Conserv. Biol.* **2013**, *28*, 76–86. https://doi.org/10.1111/cobi.12156.
