# Supplementary material for: Estimating Wolf Population Size and Dynamics by Field Monitoring and Demographic Models: Implications for Management and Conservation
Source: Animals (Basel). 2023 May 24;13(11):1735. doi: 10.3390/ani13111735 (PMC10252110; doi:10.3390/ani13111735)
Supplement: Supplementary file 1 [file animals-13-01735-s001.zip › Table S4.pdf]

SUPPLEMENTARY MATERIAL

Table S4

Self-assessment of the PVA model quality by mean of the guiding questions of Chaudhary & Oli (2020) [48].

ID	Questions	Scores
A.Background quality		
1	Were the objectives clearly specified?	1
2	Was the species life history sufficiently described?	1
3	Were the data collection techniques clearly explained?	1
4	Was the period of data collection clearly explained?	1
	Background quality comprehensive score	**1**
B. Model quality*		
5	Were the input parameters used in the PVA estimated using robust statistical methodsand were these methods described clearly?	
6	Were the uncertainties of input parameters included in the model?	
7	Was the model structure clearly defined	
8	Was the choice of the model structure clearly explained and justified?	
9	Was the model complexity and structure appropriate for the quantity and quality of data?	
10	Was the choice of the modelling platform (software package or programming language) clearly stated?	
11	Were the density-dependence effects (including Allee effect) included? If it was included, was it adequately described? If excluded was the exclusion justified?	
12	Was the environmental stochasticity included? If it was included, was it adequately described? If excluded was the exclusion justified?	

13	Was the demographic stochasticity included? If it was included, was it adequately described? If excluded was the exclusion justified?	
14	Was the genetic stochasticity included? If it was included, was it adequately described? If excluded was the exclusion justified?	
15	Was any external effect that may influence the population included, and if excluded, was the exclusion justified?	
16	Were the management scenarios (e.g. population supplementation, fire regiments, habitat restoration and predator control) included? If included were they adequately described? If excluded was exclusion justified?	
	Model quality comprehensive score	**0.85**
C. Analysis quality		
17	Were the time horizons for population projection suitable for species biology and study objectives?	1
18	Were the number of iterations used for simulations clearly stated?	1
19	Were the results of all scenarios including baseline scenario reported and discussed?	1
20	Was the study-specific definition of probability of extinction, mean time of extinction, quasi-extinction clearly stated?	NA
21	Was the mean of the population growth rate reported as an output parameter for each scenario?	1
22	Was mean of any parameter of extinction reported as an output parameter for each scenario?	0
23	Was variance of parameter of extinction reported for each scenario	1
24	Was the variance of time to extinction reported as an output parameter for each scenario?	NA
25	Was sensitivity analysis of parameter of extinction for	1

	changes in vital rates included?	
26	Was sensitivity analysis involving the time to extinction included?	NA
27	Were the limitations of model clearly discussed while making management suggestions?	1
28	Were the predictions of the model tested using follow up field data or test data?	0
	Analysis quality comprehensive score	**0.78**
**Overall quality**		**0.85**

* = because model structure was substantially derived by that of Carroll, Fredrickson & Lacy (2013) [52] whose model was evaluated in the paper by Chaudary & Oli, we adopted their score for this group;

NA = not appropriate (unuseful for the present study, since probability of extinction was negligible, i.e. 0.1%)

References

48. Chaudhary, V.; Oli, M.K. A critical appraisal of population viability analysis. *Conserv. Biol.* **2020**, *34*, 26–40. https://doi.org/10.1111/cobi.13414.

52. Carroll, C.; Fredrickson, R.J.; Lacy, R.C. Developing Metapopulation Connectivity Criteria from Genetic and Habitat Data to Recover the Endangered Mexican Wolf. *Conserv. Biol.* **2013**, *28*, 76–86. https://doi.org/10.1111/cobi.12156.
