# Supplementary material for: Estimating Wolf Population Size and Dynamics by Field Monitoring and Demographic Models: Implications for Management and Conservation
Source: Animals (Basel). 2023 May 24;13(11):1735. doi: 10.3390/ani13111735 (PMC10252110; doi:10.3390/ani13111735)
Supplement: Supplementary file 1 [file animals-13-01735-s001.zip › Table S5.pdf]

SUPPLEMENTARY MATERIAL

Table S5

Frequency of yearly success of packs detection methods (NIG = Non invasive genetics; CT = Camera trapping; WH =Wolf howling, OBS = Direct observations and carcasses)

PACK CODE	PACK NAME	NIG (n° of years)	CT (n° of years)	WH (n° of years)	OBS (n° of years)2	Pack years
1	Falterona		1	2		3
2	Muschioso-Pietra			1		1
3	Camaldoli		1	2	1	3
4	Tramignone	1	1	2		3
5	Vallesanta 1		2	2		3
6	Alto Tevere		2			2
7	Alpe di Catenaia Nord	2	3	1		3
8	Poti sud	3	3			3
9	Monte Fungaia		3		1	3
10	Scheggia	2	2			2
11	Poti Nord	3	3			3
12	Alpe della Luna Sud		3	3		3
13	Alpe della Luna Nord		2	3		3
14	Fresciano-Casteldelci		1	1	1	2
15	Sasso Simone		1	3		3
16	Pratomagno centro		2	1	1	3
17	Castel Focognano		3	1		3
18	Pratomagno sud		3	2		3
19	Monte Favalto		3			3
20	Lignano-Badicroce	3	3			3
21	Val di Chio-S.Egidio		3			3
22	Indicatore		3	1		3
23	Anghiari		1		1	1
24	Montozzi		1			1
25	Belagaio-Roccastrada				3	3
26	Civitella Paganoico-Cinigiano		1			1
27	Monterotondo-Pomarance		2		1	3
28	Civitella Paganico		2			2
29	Castellaccia-Gavorrano				3	3
30	Castiglione della Pescaia				3	3
31	Montorsaio-Rosaio Leoni	1	3	1		3
32	Arcidosso-Stribugliano		2		2	3
33	Parco della Maremma		2	2		2
34	Magliano in Toscana				3	3
35	Saturnia-Scansano 2	1	1		1	2
36	Castell'Azzara-Penna		1		1	2
37	Sorano 1		1	1	2	3
38	Sorano 2				3	3
39	Pitigliano				3	3
40	Marsiliana				3	3
41	Cavriglia			3	3	3

42	Castelnuovo Berardenga-Gaiole 2		1	2	1	3
43	Rapolano Terme-Calcione			2	3	3
44	Fontaccia-San Gimignano		3			3
45	Gambassi-Libbiano	2	3			3
46	Sovicille 1-Centrale Enel			3		3
47	Monteriggioni		1	1	1	2
48	Castelnuovo VC-Radicondoli				3	3
49	Chiusdino-Luriano		3	3		3
50	Murlo		2		1	3
51	Diga di Chianciano		2			2
52	Sarteano		2		1	3
53	Cetona		2		1	3
54	Montalcino 1		2	1		2
55	Castellina in Chianti			1	1	1
56	Pienza		1			1
57	Monti Livornesi		2	1	1	3
58	Castagneto	1	2			2
59	Caselli-Magona	2	2	1		3
60	Berignone-Tatti		2	2		3
61	Miemo		2	2	1	3
62	Chianni-Santa Luce-Casciana terme	1	1	1	1	3
63	Monterufoli		2			2
64	Montelopio				1	1
65	Fiorenzuola 3-Bruscoli				3	3
66	Fiorenzuola 1-Collinccia		1	3		3
67	Borgo San Lorenzo-Scarperia-Mansalto	2	2	1		3
68	Palazzolo sul Senio-Roncaccio	2	3	1		3
69	Fiorenzuola 2-Piancaldoli				3	3
70	Marradi-Gamogna		1		2	3
71	Vicchio-Peschiena	1	1	1	1	3
72	Carbonile				1	1
73	Monte Giovi-Pontassieve		2		1	3
74	P.Pratone-Fiesole-Pontassieve				3	3
75	Incisa-S.Polo			3		3
76	p.Conca-Vaglia-Borgo San Lorenzo		2		1	3
77	Barberino Mugello-Firenzuola-Gazzarro		1		2	3
78	Ronta				1	1
79	Gualdo-Pomino	1		2		2
80	Dudda			1		1
81	Montaione-Pillo	2	2		1	3
82	Montanino-Reggello		1	1	1	3
83	Badia				2	1

84	Barberino Val d'Elsa-Poggibonsi-Castellina				2	2
85	Calvana				1	1
86	Luicciana-Cantagallo		1		2	3
87	Carigiola			1		1
88	Lavello-Acquerino		1	2		3
89	Sambuca Pistoiese		1	2		3
90	Lizzo-Castel di casio			2	1	3
91	Torri-Cantagallo-Sambuca	1	3	1		3
92	Brana-Sambuca p.se			2	1	3
93	Croce Arcana-Volata				3	3
94	Dynamo-S.Marcello P.se		1	2	1	3
95	Calamecca-Pontito			1	2	3
96	Marliana-Nievole		2	1	1	3
97	Apuane Sud				3	3
98	Apuane Nord	1	2		1	3
99	Orecchiella		2	2	1	3
100	Capraia-Fosciandora			1	2	3
101	Orrido di Botri				2	2
102	Renaio-Monte Giovo				2	2
103	Zeri 1			3		3
104	Zeri 2			3		3
105	Pontremoli			3		3
106	Filattiera			3		3
107	Bagnone-Licciana nardi			3		3
108	Fivizzano		1	3		3
109	Casola-Giuncugnano			3		3
110	Villafranca		2			2
111	Abetone-Orrido di botri				3	2
112	Uccelliera				2	2
113	San Marcello Pistoiese				2	2
114	Calenzano		2			2
115	Sovicille 2		1	1	1	2
116	Montalcino 2				1	1
117	Abbadia				1	1
118	Asciano				1	1
119	Gaiole 1		1	1		2
120	Cotorniano		1	1		1
121	Siena				1	1
122	Castelnuovo Berardenga		1	2		2
123	Montieri 1				1	1
124	Montieri 2				1	1
125	Manciano		1			1
126	Scansano 1		1		1	2
127	Semproniano-Rocconi				2	2
128	Amiata				1	1
129	Cinigiano				1	1
130	Alpe di Catenaia Nord-Dama		1		1	1
131	Vallesanta 2		1	1		1
132	Radicofani			2		2

133	San Quirico d'Orcia				1	1
134	Follonica-Suvereto-Montioni		3			3
135	Radda			2		2
N° of pack-years monitored with each method		**32**	**145**	**113**	**120**	**326**
% on the 326 pack-years		9.8	44.5	34.7	36.8	
N° of packs monitored with each method		19	80	63	74	
N° of packs monitored with only one method		0	21	14	29	
