# Supplementary figures and images for: Estimating Wolf Population Size and Dynamics by Field Monitoring and Demographic Models: Implications for Management and Conservation
Source: Animals (Basel). 2023 May 24;13(11):1735. doi: 10.3390/ani13111735 (PMC10252110; doi:10.3390/ani13111735)

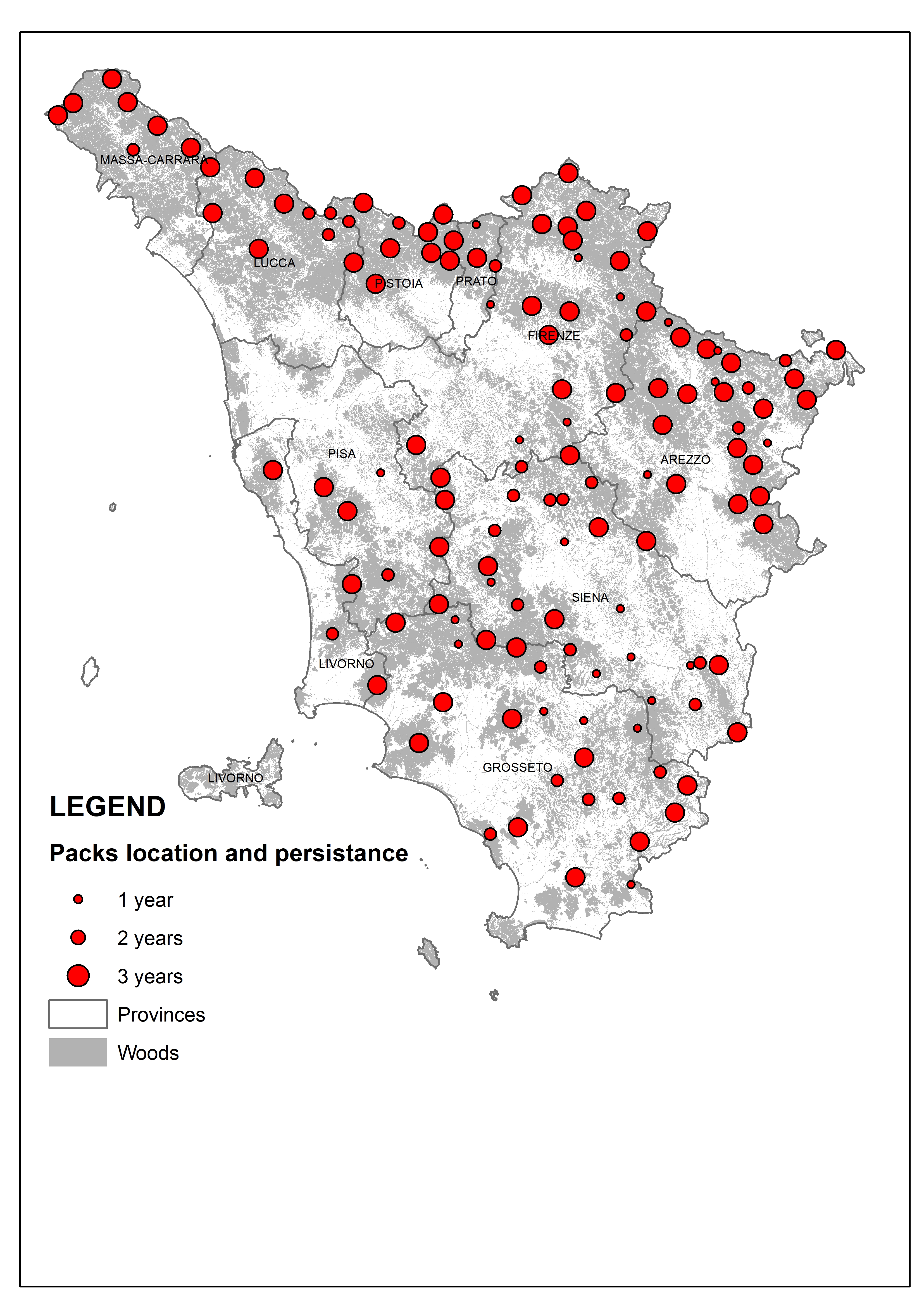

Supplement: Supplementary file 1 [file animals-13-01735-s001.zip › Figure S1.jpg]

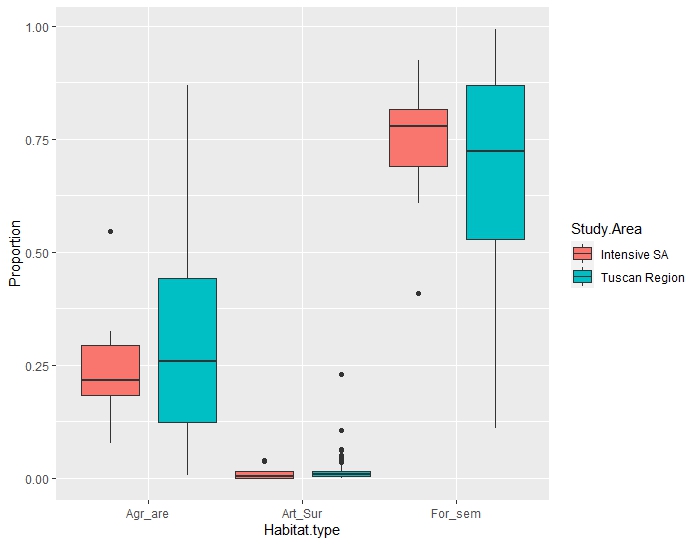

Supplement: Supplementary file 1 [file animals-13-01735-s001.zip › Figure S2a.jpeg]

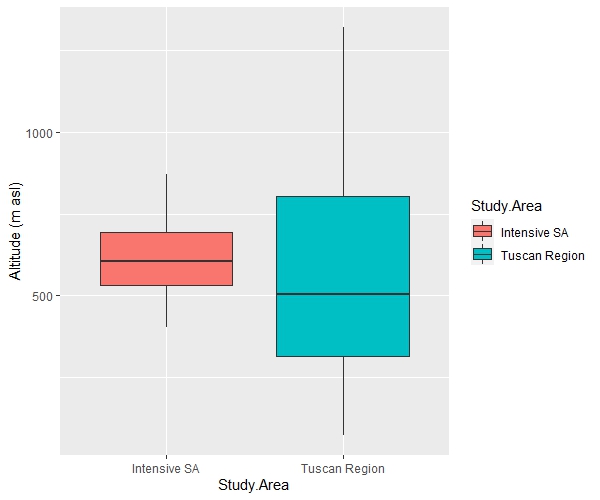

Supplement: Supplementary file 1 [file animals-13-01735-s001.zip › Figure S2b.jpeg]

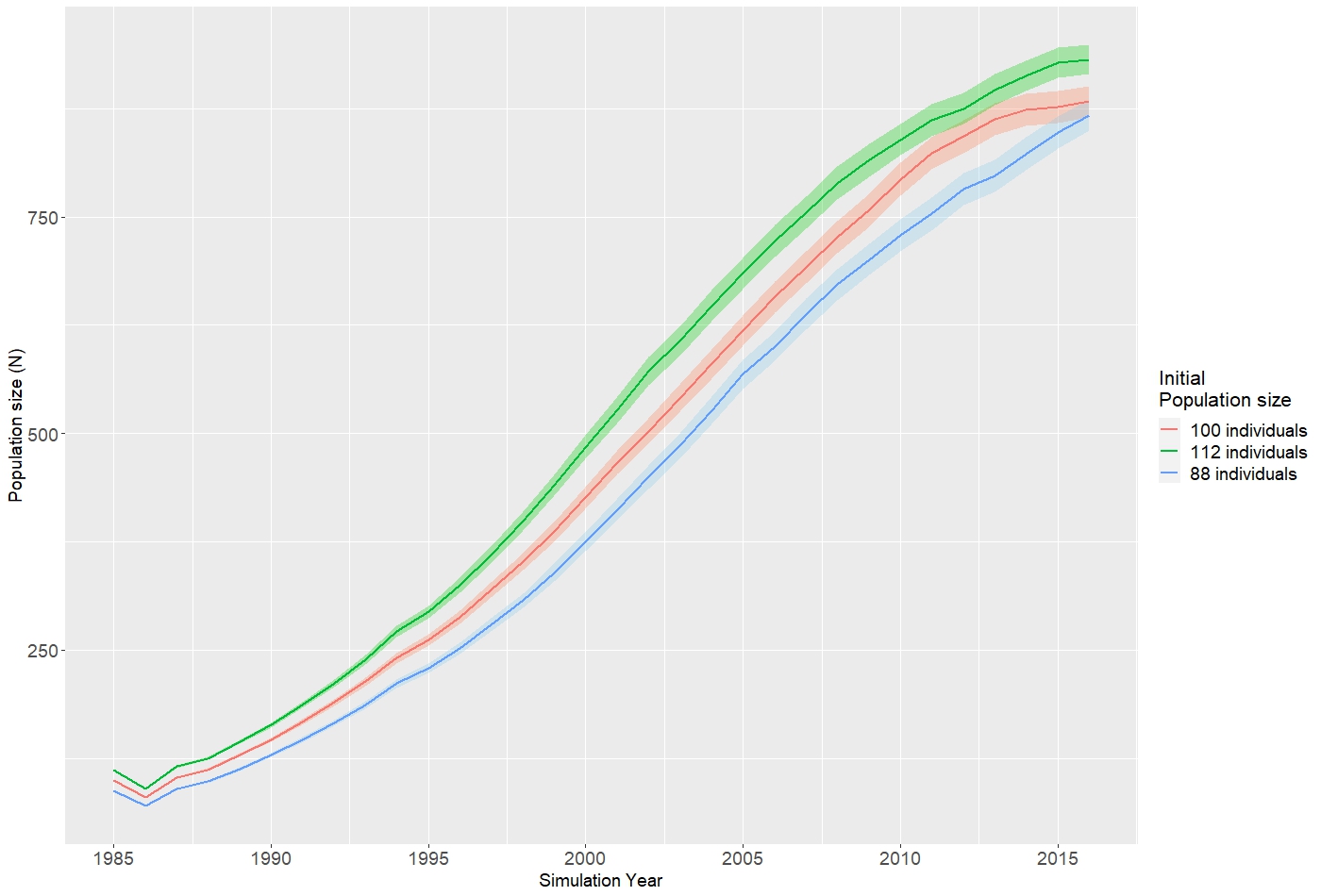

Supplement: Supplementary file 1 [file animals-13-01735-s001.zip › Figure S3.jpeg]
